# Strawberry Phytochemicals Inhibit Azoxymethane/Dextran Sodium Sulfate-Induced Colorectal Carcinogenesis in Crj: CD-1 Mice

**DOI:** 10.3390/nu7031696

**Published:** 2015-03-10

**Authors:** Ni Shi, Steven K. Clinton, Zhihua Liu, Yongquan Wang, Kenneth M. Riedl, Steven J. Schwartz, Xiaoli Zhang, Zui Pan, Tong Chen

**Affiliations:** 1 Division of Medical Oncology, Department of Internal Medicine, The Ohio State University, Columbus, OH 43210, USA; E-Mails: ni.shi@osumc.edu (N.S.); steven.clinton@osumc.edu (S.K.C.); 2 Comprehensive Cancer Center, The Ohio State University, Columbus, OH 43210, USA; E-Mails: kenneth.riedl@osumc.edu (K.M.R.); steven.schwartz@osumc.edu (S.J.S.); 3 State Key Laboratory of Molecular Oncology, Cancer Institute and Hospital, Chinese Academy of Medical Sciences, Beijing 100021, China; E-Mails: zhl60@yahoo.com (Z.L.); twang5511@yahoo.com (Y.W.); 4 Department of Food Science and Technology, The Ohio State Univeristy, Columbus, OH 43210, USA; 5 Center for Biostatistics, The Ohio State University, Columbus, OH 43210, USA; E-Mail: xiaoli.zhang@osumc.edu; 6 Division of Cardiovascular Medicine, Department of Internal Medicine, The Ohio State University, Columbus, OH 43210, USA; E-Mail: zui.pan@osumc.edu

**Keywords:** strawberry, phytochemicals, chemoprevention, colorectal cancer, colitis

## Abstract

Human and experimental colon carcinogenesis are enhanced by a pro-inflammatory microenvironment. Pharmacologically driven chemopreventive agents and dietary variables are hypothesized to have future roles in the prevention of colon cancer by targeting these processes. The current study was designed to determine the ability of dietary lyophilized strawberries to inhibit inflammation-promoted colon carcinogenesis in a preclinical animal model. Mice were given a single i.p. injection of azoxymethane (10 mg kg^−^^1^ body weight). One week after injection, mice were administered 2% (w/v) dextran sodium sulfate in drinking water for seven days and then an experimental diet containing chemically characterized lyophilized strawberries for the duration of the bioassay. Mice fed control diet, or experimental diet containing 2.5%, 5.0% or 10.0% strawberries displayed tumor incidence of 100%, 64%, 75% and 44%, respectively (*p* < 0.05). The mechanistic studies demonstrate that strawberries reduced expression of proinflammatory mediators, suppressed nitrosative stress and decreased phosphorylation of phosphatidylinositol 3-kinase, Akt, extracellular signal-regulated kinase and nuclear factor kappa B. In conclusion, strawberries target proinflammatory mediators and oncogenic signaling for the preventive efficacies against colon carcinogenesis in mice. This works supports future development of fully characterized and precisely controlled functional foods for testing in human clinical trials for this disease.

## 1. Introduction

Colorectal cancer (CRC) remains the third most common cause of cancer-related death in the United States and the fourth globally with a rising incidence, which is in parallel with economic development and adoption of an affluent diet and lifestyle [1,2]. A wealth of research in experimental models and humans implicates inflammatory processes as a risk factor for colorectal carcinogenesis [3]. Inflammatory bowel disease (IBD) represents an assortment of chronic inflammatory syndromes that greatly increases the risk for developing CRC, and is proportional to the severity, extent and duration of disease [4]. Unlike sporadic CRC, which is characterized by adenomatous polyps that can be detected through routine colonoscopy and removed by endoscopic polypectomy, colitis-associated CRC arises from focal or multifocal patchy and flat dysplasia in areas of inflammation, which are more difficult to identify during surveillance colonoscopy. Experimental models of colitis-associated CRC can help elucidate how chronic inflammation, its various cellular and cytokine mediators, mediate the initiation and progression of CRC.

The protective roles of diets rich in fruits and vegetables have been previously reported [2]. Strawberries have been extensively evaluated for their impact on human health in past years due to their rich phytochemical profiles, efficacy in rodent models and minimal or no toxicity observed in pilot human studies [5,6]. In rodent models, consumption of strawberries has shown anticancer activity in oral cavity [7], breast [8], lung [9] and esophagus [10]. These observations led us to undertake a Phase II clinical investigation for those diagnosed with esophageal dysplasia. We demonstrated that dietary intake of strawberries (60 g day^−^^1^ for 6 months) inhibited the progression of precancerous lesions [11]. Our earlier work in the human esophagus is suggestive of anti-inflammatory activity of strawberry components via suppression of nuclear factor kappa B (NFκB) activation, down-regulation of cyclooxygenase-2 (COX-2) and inducible nitric oxide synthase (iNOS) [11].

In recent years, a critical role of epidermal growth factor (EGF) signaling in CRC has emerged [12]. EGF receptor (EGFR) pathways implicated in CRC include the RAS/RAF/mitogen-activated protein kinases (MAPK)/Extracellular signal-regulated kinases (ERK) and the phosphatidylinositol 3-kinase (PI3K)/Akt/PTEN/mammalian target of rapamycin (mTOR) pathways, which are involved in the activation of NFκB [13,14]. The MAPK pathway (ERK, c-Jun-NH2 kinase and p38) represents a superfamily of proteins that regulate cell differentiation, proliferation and survival during carcinogenesis [15]. During inflammatory colitis, the activation of PI3K/Akt mediates phosphorylation of p65 NFκB at Ser^536^, which leads to the activation of NFκB and release of proinflammatory cytokines [16]. The activation of NFκB is detected in murine models and more than 50% of human CRC cases [17,18]. NFκB regulates multiple genes that drive inflammatory responses, including COX-2 and iNOS, which can promote the development of CRC. Thus, examination of the signaling pathways downstream of EGFR is central to understand the progression of CRC.

In the current study, we are particularly interested in the anti-carcinogenetic activity of strawberries (*Fragaria x ananassa*) and their constituents against CRC. The present study addresses the hypothesis that strawberries will inhibit azoxymethane (AOM)/dextran sodium sulfate (DSS)-induced colon carcinogenesis in murine model. We further explore the mechanisms of action of strawberries on inflammation and signaling processes that may mediate the anticancer activities.

## 2. Materials and Methods

### 2.1. Chemicals and Reagent Kits

AOM was obtained from Sigma Chemical Co. (St. Louis, MO, USA). DSS (molecular weight 36,000–50,000) was purchased from ICN Biochemicals (Aurora, OH, USA). The nitrotyrosine antibody was supplied by Millipore (Billerica, MA, USA), and other immunohistochemistry staining reagents were supplied by BioGenex (Fremont, CA, USA). The Fast SYBR Green Master Mix Kit was obtained from Applied Biosystems (Grand Island, NY, USA). The antibodies specific for p65 NFκB, p-p65 NFκB (Ser^536^); PI3K, p-PI3K (Tyr^458^); Akt, p-Akt (Thr^308^), ERK1/2 and p-ERK1/2 (Thr^202^/Tyr^204^) were purchased from Cell Signaling Technology (Danvers, MA, USA). The DC Protein Assay Kit and Immun-star™ WesternC™ Kit were obtained from Bio-Rad Laboratories, Inc. (Hercules, CA, USA). The PGE_2_ ELA Kit and Nitrate/Nitrite Colorimetric Assay Kit were supplied by Cayman Chemical Co. (Ann Arbor, MI, USA).

### 2.2. Lyophilized Strawberries

Lyophilized strawberries (*Fragaria x ananassa*) were obtained from the California Strawberry Commission. The strawberries used in this study contained University of California cultivars (Camarosa and Ventana) and one proprietary cultivar, Well-Pict #269. Since the lyophilized (freeze-dried) strawberries are not mechanically or chemically sterilized, the material was stored in sealed bags (20 lb aliquots) in a −20 °C freezer at all times. The lyophilized strawberries were added to AIN-76A diet at the concentrations of 2.5%, 5% or 10% by weight, respectively. The experimental diet, therefore, contained 2.5%, 5% or 10% strawberries and was prepared fresh weekly and stored at 4 °C until fed as described in the previous similar study [19]. Berries were analyzed for content of certain vitamins, minerals, phenols, carotenoids, and phytosterols by Covance Laboratories (Madison, WI, USA) as described previously [11]. We have shown that the quantity of major anthocyanins and 26 micronutrients in freeze-dried berries vary by only 10%–20% over a 5-year period when stored at −20 °C [20]. In our recent clinical study with strawberries from the same source, we obtained berries over 3 consecutive growing seasons. During our ongoing and previous studies, berry phytochemical consistency of < +/−10% was observed [11].

### 2.3. HPLC-MS/MS Analysis of Strawberry Phytochemicals

To characterize the bioactive components in strawberry powder that was used in this animal study, strawberry phytochemicals were identified with a combination of high performance liquid chromatography/tandem mass spectrometry (HPLC-MS/MS), accessible standards, UV-vis and reported mass as described previously [21].

#### 2.3.1. Extraction

Sample (100 mg) of lyophilized strawberries was extracted twice using 5 mL of acidified acetone (acetone:water:formic acid, 76:19:5) with brief probe sonication (5 s) and left to extract for 20 min sonicated (Sonic Dismembrator 150E, Fisher Scientific, Pittsburgh, PA, USA) at 23 °C and centrifuged at 2000× *g* for 1 min under ambient conditions. Supernatants were pooled, and extracts were dried under nitrogen gas. Residues were re-dissolved in acidified 20% methanol (water:methanol:formic acid, 76:19:5), and filtered (2 μm, 13 mm, polytetrafluoroethylene, Whatman Laboratory Division, Clifton, NJ, USA) prior to analysis.

#### 2.3.2. Identification

For identification of strawberry phytochemicals, HPLC (2695 HPLC, Waters, Milford, MA, USA) instrumentation equipped with column heater set at 35 °C, autosampler, PDA detector (Waters Corp, Milford, MA, USA) was combined with a Waters Q-Tof Premier (Micromass MS Technologies, Manchester, UK). Zorbax SBCN (250 × 4.6 mm, 5 μm, Santa Clara, CA, USA) was used for separation and the mobile phase consisted of 1% (v/v) aqueous formic acid (A) and 1% (v/v) formic acid in acetonitrile (B) at 1.3 mL min^−1^. The gradient began at 100: 0 (A: B) increased linearly to 75:25 over 40 min, 15:85 at 42 min, hold for 2 min, re-equilibrate to initial conditions over 3 min. The HPLC flow was split 1:10 prior to the MS. Injection volume was 20 μL. Positive and negative ion modes were used for MS analysis having capillary voltage of 3.2 kV and 2.8 kV, respectively. Sodium formate was used for calibration of the MS in the range of 50–3000 *m*/*z*. Leucine enkephalin was used as lockSpray mass with *m*/*z* at 556.2771^+^/554.2615^−^. Dry gas flow was at 700 L h^−1^, cone voltage at 35 V and desolvation gas temperature was at 480 °C.

Standards of pelargonidin-3-*O*-glucoside and ellagic acid were used to confirm their identities. Other compounds were identified based on accurate mass, UV-vis features and literatures precedent for strawberries. Anthocyanins were identified based on anthocyanidin fragments (cyanidin *m*/*z* 287, pelargonidin *m*/*z* 271) and UV-vis (520 nm for cyanidin, 500 nm for pelargonidin). Quercetin and kaempferol glycosides were identified by characteristic fragments of *m*/*z* 301.035 and *m*/*z* 285.040, respectively, as well as 340–355 nm spectral features. Ellagitannins were identified based on m/z 300.999 ellagic acid fragments and 260 nm UV feature. Ellagic acid and ellagic acid derivatives were identified based on *m*/*z* 300.999 parent or fragment and 365 nm UV feature.

#### 2.3.3. Quantification

Phenolics in strawberries were quantified using the HPLC of the same LCMS system described above monitoring at 260, 355, and 500 nm. Chromatographic conditions for quantification were the same as those used for identification. Injection volume was 20 µL. Authentic ellagic acid and cyanidin-3-*O*-glucoside standards were used for quantification of strawberry phenolics (ellagic acid, ellagitannin and pelargonidin). Calibration curves were performed by injecting the standards three times at five different concentrations 5 to 100 μmol for cyanidin-3-*O*-glucoside and 0.5 to 10 µmol for ellagic acid (*R*^2^ > 0.9990). Pelargonidin was quantified using chromatographic peak areas recorded at 500 nm using pelargonidin-3-*O*-glucoside standard curve and published extinction coefficients [22]. Ellagic acid and ellagic acid derivatives were expressed in ellagic acid equivalents. Ellagitannin were quantified based on ellagic acid using previously published methods [23].

### 2.4. Experimental Procedure

This study was approved by Institutional Animal Care and Use Committee (60013170, 2008). Male Crj: CD-1 (ICR) mice were purchased from Taconic Farm Inc. After a 2-week period of acclimation to the animal facility, mice (7-week old) were randomly assigned to five experimental groups (10 mice each group) and placed on an AIN-76A diet (Table 1). At the beginning of the study, mice in Group 1 were given a single intraperitoneal (i.p.) injection of saline (vehicle for AOM) and mice in Groups 2 to 5 were given a single i.p. injection of AOM (10 mg kg^−1^ body weight). Starting 1 week after injection, mice in Groups 2–5 were administered 2% (w/v) DSS in drinking water for 7 days. Beginning at week 4, mice in Groups 1 and 2 were kept on a standard AIN-76A diet and mice in Groups 3 to 5 were fed AIN-76 diet containing 2.5%, 5% or 10% lyophilized strawberries for the duration of the bioassay. The diet and water were available *ad libitum*. Food consumption and body weight were measured weekly. At 20 weeks, animals were sacrificed and subjected to gross necropsy. The colon of each animal was collected, flushed with ice-cold phosphate-buffered saline (PBS), excised and opened longitudinally. The entire colon tissue was macroscopically inspected and was cut into halves longitudinally. Tissues were fixed in 10% neutral formalin or frozen in liquid nitrogen immediately.

### 2.5. Evaluation of Histological Grade

The H & E stained slide were viewed and photographed with a dual-head Nikon microscope with a high-resolution spot camera interfaced with computer-loaded image analysis software (Simple PCI Imaging Systems; Compix, Inc., Cranberry Township, PA, USA). Each viewing field was categorized into one of four histological categories: normal, dysplasia, adenoma and adenocarcinoma. The classification scheme used was a modification of criteria developed by Riddell *et al.* and Pascal, with consideration of pathology of mouse models given by Boivin *et al.* [24,25,26]. In brief, the normal colonic epithelium comprises of a single layer of columnar epithelium, intact lamina propria and an outer layer of lamina muscularis mucosae. Colonic mucosal dysplasia is characterized by elongated, crowded and pseudostratified nuclei. Adenoma is defined as a protrusion that develops from surface epithelium of the colon exhibiting a serrated architecture with evidence of dysplasia. Colonic adenocarcinoma is characterized by hyperchromatic nuclei, a higher nuclear to cytoplasmic ratio, loss of polarity, varied cellular shapes, and invasion into the lamina propria or muscularis.

**Table 1:** Effect of strawberries on AOM/DSS-induced colorectal cancer (CRC) in mice.

Group	Treatment	Diet	Tumor Incidence (%)	Tumor Multiplicity (Mean ± SE)	Histologic Grade (Mean ± SE; %)			
					Normal	Dysplasia	Adenoma	Adenocarcinoma
1	None	AIN-76A	0	0	-	-	-	-
2	AOM ^a^ + DSS ^b^	AIN-76A	100	3.9 ± 1.5	20.2 ± 3.7	29.7 ± 3.2	20.8 ± 8.1	28.8 ± 12.1
3	AOM + DSS	2.5% Straw ^c^	64	3.8 ± 1.4	38.5 ± 9.1 ^f^	46.0 ± 2.3 ^f^	10.6 ± 6.4 ^g^	4.7 ± 4.6 ^g^
4	AOM + DSS	5.0% Straw	75	3.0 ± 1.1	40.1 ± 4.0 ^f^	50.0 ± 5.0 ^f^	6.2 ± 5.0 ^g^	4.7 ± 4.7 ^g^
5	AOM + DSS	10.0% Straw	44 ^d^	1.8 ± 1.0 ^e^	45.7 ± 4.0 ^f^	48.7 ± 1.3 ^f^	2.8 ±2.8 ^g^	2.8 ± 2.8 ^g^

^a^ AOM = azoxymethane; 10.0 mg kg^−^^1^ body weight, single i.p. injection. ^b^ DSS = dextran sodium sulfate; 2% (w/v) in drinking water for 7 days. ^c^ Straw = chemically characterized lyophilized strawberries. ^d^ Significantly lower than Group 2 as determined by χ*^2^* test (*p* < 0.05). ^e^ Significantly lower than Group 2 as determined by analysis of variance (*p* < 0.05). ^f^ Significantly higher than Group 2 as determined by linear mixed effect model (*p* < 0.05). ^g^ Significantly lower than Group 2 as determined by linear mixed effect model (*p* < 0.05).

### 2.6. Modified Disease Activity Index

DAI is commonly used by researchers to assess disease condition in studies of colitis in preclinical models [27]. Based on our observations during the acute colitis and carcinogenesis phases, we found that soft stool and bleeding are two major symptoms in mice. We modified the DAI measurement in this study to assess the two parameters—stool consistency and presence of fecal blood. Evaluations of disease activity were performed daily. The DAI score was calculated using the following 4-point scale: 0, normal; 1, soft stool without visible blood; 2, visible blood in stool; and 3, soft stool with visible blood.

### 2.7. Immunohistochemistry

Formalin-fixed and paraffin-embedded blocks were serially sectioned at 4 μm and mounted on SuperFrost Plus slides (Fisher Scientific, Pittsburgh, PA, USA). In brief, after deparaffinization and antigen retrieval, slides were immersed in 3% hydrogen peroxide for 20 min to block endogenous peroxidase activity followed by rinsing with wash buffer. After the nonspecific binding was blocked, sections were incubated with rabbit polyclonal anti-nitrotyrosine (1:600) for 1 h at room temperature followed by 20 min incubation with secondary antibody using a rabbit adsorbed link and strepavidin-horseradish peroxidase label. The sections were developed with diaminobenzidine chromogen, and then counterstained with hematoxylin, dehydrated, and mounted. For the negative control, the primary antibody was replaced with PBS and normal serum. The stained slides were viewed and photographed with Nikon Eclipse 80i microscope. The nitrotyrosine positive staining cells were counted using NIS-element software (Nikon) under at least 5 randomly-selected views. To determine the score of nitrotyrosine staining, the following 5-grade system was applied: Grade 0, no immunoreactivity (no positive cells); Grade 1, weak immunoreactivity (≤10% positive cells); Grade 2, mild immunoreactivity (11%–30% positive cells); Grade 3, moderate immunoreactivity (31%–60% positive cells); and Grade 4, strong immunoreactivity (61%–100% positive cells) [28].

### 2.8. Real-Time Polymerase Chain Reaction

Total cellular RNA was isolated from frozen tissues and reverse transcription was performed using High-Capacity cDNA Reverse Transcription Kits as described previously [11]. Real-time PCR was performed in a 7900HT Fast Real-Time PCR System (Applied Biosystems, Grand Island, NY, USA) using the Fast SYBR Green Master Mix Kit. The primers for real-time PCR were list in Supplementary Table S1. The amplification results were analyzed using sequence detection system (SDS) 2.2.1 software (Applied Biosystems, Grand Island, NY, USA). The genes of interest were normalized to the corresponding GAPDH. Data were expressed as fold-change in mice fed experimental diet relative to mice fed control diet.

### 2.9. Western Blot Analysis

Proteins were extracted from frozen tissues and their concentrations were measured using a DC Protein Assay Kit as described previously [11]. The PVDF membrane was blocked and incubated overnight at 4 °C with specific primary antibodies followed with secondary antibody for 1 h. Anti-GAPDH antibody was used as an internal reference. The immunoreactive bands detected by Immun-star™ WesterC™ Kit were developed by Molecular Imager ChemiDoc XRS (Bio-Rad Laboratories, Hercules, CA, USA) and quantified using Image Lab Software (Version 2.0, Bio-Rad Laboratories). The membranes were stripped using Restore™ Western Blot Stripping Buffer (Thermo Scientific, Waltham, MA, USA) between probing with different antibodies.

### 2.10. Prostaglandin E_2_ Measurement and Nitrate/Nitrite Colorimetric Assay

The PGE_2_ concentration and iNOS activity were detected using a PGE_2_ ELA Kit and Nitrate/Nitrite Colorimetric Assay Kit, respectively, as described previously [19].

### 2.11. Statistical Analysis

Body weight, food consumption, tumor multiplicity, DAI measurements, expression levels of p-p65 NFκB, p-PI3K, p-Akt, p-ERK1/2, COX-2, iNOS, TNF-α, IL-1β and IL-6, PGE_2_ and nitrite/nitrate productions as well as scores for nitrotyrosine immunoreactivity were analyzed and compared using one-way analysis of variance (ANOVA). Tumor incidence data were analyzed using the χ*^2^* test. For histopathological analysis, the number of field of each tissue type was first normalized to the total number of field in each animal, and then a linear mixed effects model was used to analyze the correlations between AOM/DSS control animals and animals fed experimental diets. Holm’s procedure was used to adjust for multiple comparisons to strongly controlled type I error at 0.05 [29]. All statistical analysis was carried out using SAS 9.2 (SAS Institute Inc., Cary, NC, USA).

## 3. Results

### 3.1. Characterization and Quantification of Phytochemicals in Strawberries

**Figure 1:**
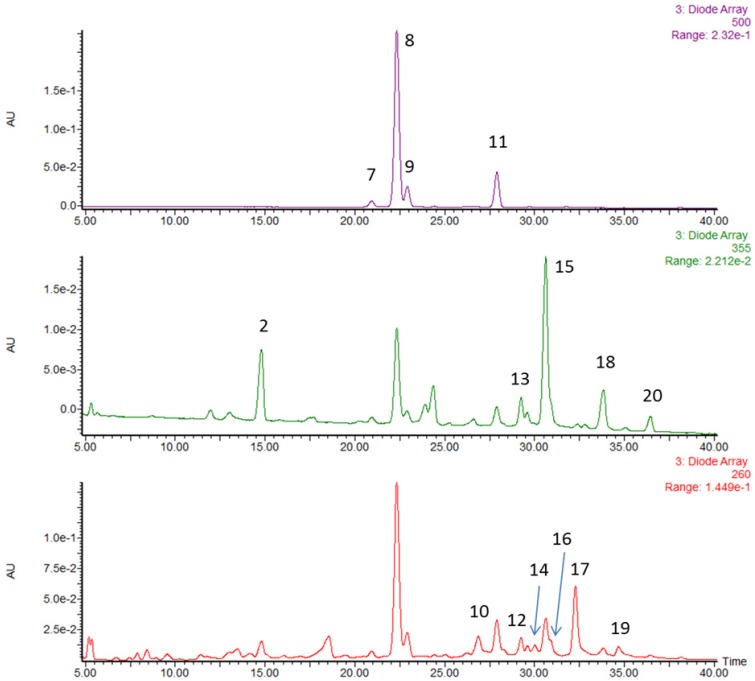
High performance liquid chromatography (HPLC) chromatogram of strawberry extract separated on Zorbax SBCN 4.6 × 250 mm, 5 µm column with 1% (v/v) formic acid in water *versus* 1% (v/v) formic acid in acetonitrile mobile phase gradient. The peak identities were established using accurate mass MS results as well as UV-vis features and literature precedent for strawberry analysis.

In order to identify the individual components responsible for the anti-inflammatory and anti-cancer activities in strawberry powder, we chemically characterized the main phytochemicals by HPLC-MS/MS. The same HPLC run was displayed at three wavelengths (260, 355 and 500 nm), those at which the various species were quantified (Figure 1). The peak identifications and quantitative results are listed in Table 2. We identified twenty phytochemicals and quantified fourteen of them. Peaks 1 to 6 were identified by MS but not detectable by diode array. Peaks 6, 10, 12 and 14 have the same exact mass and are isomers of the same ellagitannin. The fourteen quantified compounds are classified to three categories: anthocyanins (58.4%, w/w of the phenolics), ellagitannin/ellagic acid/ellagic acid derivatives (16.9%) and flavonols (10.5%). Anthocyanins are the main polyphenolic in strawberries including pelargonidin glucoside (41.1%), pelargonidin malonyl glucoside (9.38%), pelargonidin rutinoside (6.19%) and cyanidin glucoside (1.67%).

### 3.2. General Observations

**Figure 2:**
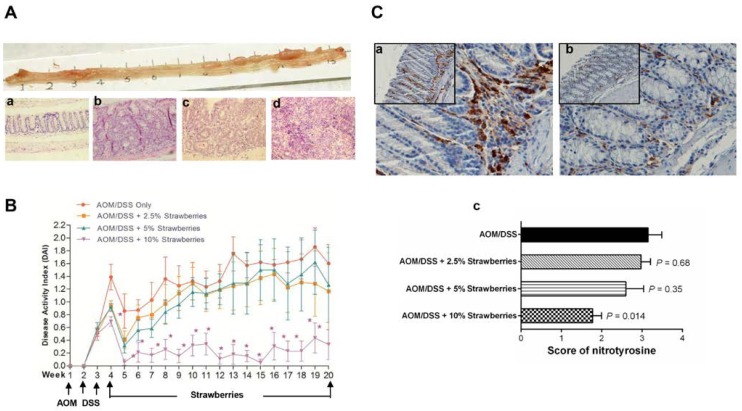
(**A**) upper panel, representative macroscopic appearance of mouse colon treated with azoxymethane (AOM)/dextran sodium sulfate (DSS). Lower panel, representative hematoxylin and eosin stained sections show different histopathology of mouse colon including normal (a), dysplasia (b), adenoma (c) and adenocarcinoma (d). Images are shown at 200× magnification; (**B**) disease activity index (DAI) of AOM/DSS-treated mice. Mice fed strawberries had lower DAI than those fed control diet; bars, ± SE. * *p* < 0.05; and (**C**) immunochemistry of nitrotyrosine. Stronger positive nitrotyrosine immunoreactivity infiltrated in the lamina propria was observed in mice fed control diet (a) compared to those fed strawberries (b). Strawberries decreased scores of nitrotyrosine immunoreactivity (c).

Body weights and food consumption (~5 g day^−1^) in all animals were not significantly different among dietary groups throughout the bioassay. Bloody and soft stools were observed one week after DSS treatment. These symptoms improved after 5 weeks from the initiation of AOM/DSS. The representative macroscopic appearances of colon in mice treated with AOM/DSS are shown in Figure 2A.

**Table 2:** Bioactive phenolic compounds identified in lyophilized strawberries.

Peak No.	RT (min)	[M–H]^−^ MSel ^a^	[M–H]^−^ MSe2 ^b^	Peak Assignment	mg (100 g)^−1^	% by Weight
1	13.0	783.1	301.0	ellagitannin	-	-
2	14.8	325.1	145	coumaroyl hexoside	-	-
3	15.2	577.1	289	procyanidin dimer	-	-
4	16.1	289.1	-	catechin	-	-
5	17.1	577.1	289	procyanidin dimer	-	-
6	18.0	633.1	301.0	ellagitannin	-	-
Anthocyanins						
7	20.9	447.1	285	cyanidin glucoside	14.9	1.7
8	22.3	431.1	269	pelargonidin glucoside	367.7	41.1
9	22.9	577.2	269	pelargonidin rutinoside	55.3	6.2
11	27.9	473	269	pelargonidin malonyl glucoside	83.9	9.4
					Total	58.4
Ellagitannin/ellagic acid/ellagic acid derivatives						
10	26.9	935.1	301.0	ellagitannin	64.1	7.2
12	28.3	935.1	301.0	ellagitannin	11.4	1.3
13	29.3	447.1	301.0	ellagic acid rhamnoside	23.1	2.6
14	30	935.1	301.0	ellagitannin	23.1	2.6
16	30.7	301.0	-	ellagic acid	7.3	0.8
17	32.3	(934.1)^2−^	301.0	agrimoniin	144.5	16.2
19	34.6	(1401.6)^2−^	301.0	lambertianin	20.3	2.3
					Total	16.9
Flavonols						
15	30.6	477.1	301.0	quercetin hexuronide	58.8	6.6
18	33.8	447.1	285	kaempferol glucoside	14.5	1.6
		461.1		kaempferol hexuronide		
20	36.4	489.1	285	kaempferol malonyl hexoside	5.1	0.6
					Total	10.5

^a^ MSe1 = MS scans with low collision energy; ^b^ MSe2 = MS scans with high collision energy to induce fragmentation and produce MS/MS spectra.

### 3.3. Strawberries Inhibit Tumor Development and Reduce the Disease Activity Index

At week 20, tumor incidence decreased from 100% in mice fed the control diet to 64%, 75% and 44% in mice fed 2.5%, 5% and 10% strawberries, respectively (Table 2). In parallel, tumor multiplicity was reduced from 3.9 ± 1.5 tumors per mouse in AOM/DSS-treated mice to 3.8 ± 1.4, 3.0 ± 1.1 and 1.8 ± 1.0 tumors per mouse in mice fed 2.5%, 5% and 10% strawberries, respectively. The portions (%) of normal, dysplasia, adenoma and adenocarcinoma in animals fed different experimental diets are shown in Table 2. Statistical analysis showed that dietary administration of strawberries significantly increased the proportions of fields appearing normal and dysplasia, whereas the proportions of fields appearing adenoma and adenocarcinoma were decreased in strawberry treatment groups compared to AOM/DSS control group. In addition, the trend of proportion changes in each tissue type was associated with the strawberry doses (*p* < 0.05).

We measured DAI during the entire complete-carcinogenesis bioassay including stages of acute colitis and tumor formation. As shown in Figure 2B, bloody and soft stools (caused by acute colitis) were observed during the 1st week of DSS treatment and these symptoms improved during the following 1–2 weeks. Bloody and soft stools (caused by tumor development) occurred from week 4. Dietary strawberries improved the symptoms during week 4 to week 20 compared to mice given AOM/DSS only.

### 3.4. Strawberries Reduce Nitrotyrosine Production

We detected the immunoreactivity of nitrotyrosine, a proposed index of nitrosative stress. As shown in Figure 2C, the nitrotyrosine staining showed more numerous and concentrated positive cells in mice treated with AOM/DSS fed control diet when compared to mice fed strawberries. Staining score was reduced 6%, 17%, and 44% (*p* = 0.014) by feeding 2.5%, 5.0% or 10.0% strawberry powder, respectively.

### 3.5. Strawberries Decrease Phosphorylation of PI3K, Akt, ERK and NFκB

**Figure 3:**
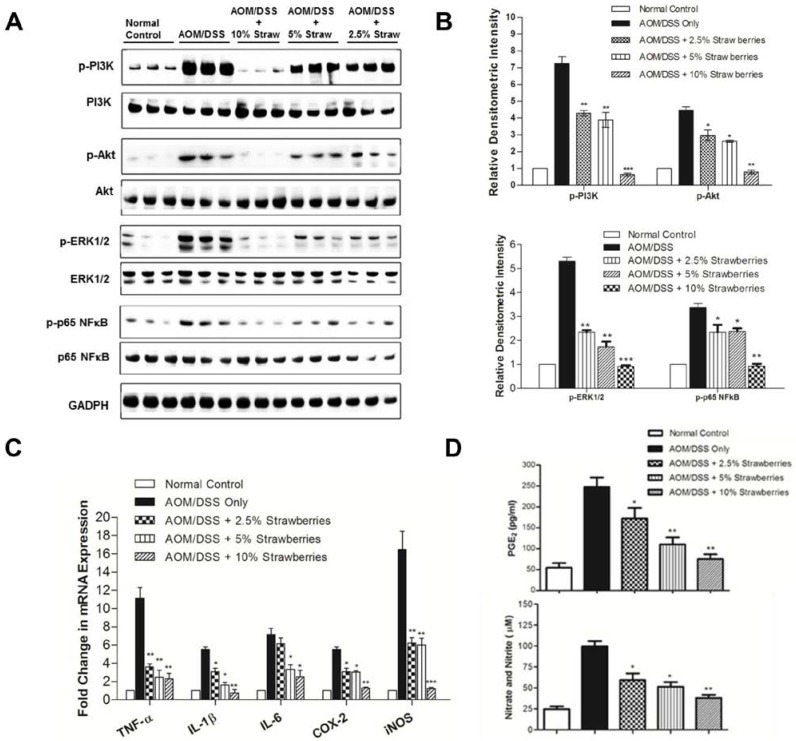
Western blot analysis of effects of strawberries on PI3K/Akt, ERK and NFκB (A,B). Strawberries decrease phosphorylation of PI3K/Akt, ERK1/2 and activation of NFκB. Samples were probed with antibodies against p-PI3K, p-Akt, p-ERK1/2 or p-p65 NFκB. The Western blot membranes were stripped and reprobed for GADPH as an internal control to confirm equal loading. (**A**) representative blots from one of three separate experiments; (**B**) relative densitometric intensity; (**C**) real-time PCR analysis of the inhibitory effects of strawberries on mRNA expression of TNF-α, IL-1β, IL-6, COX-2 and iNOS; and (**D**) inhibitory effects of strawberries on the production of PGE_2_ and NO. *Columns*, mean; *bars*, ± SE; * *p* < 0.05, ** *p* < 0.01, *** *p* < 0.001 *versus* animals treated with AOM/DSS fed control diet.

To determine whether AOM/DSS treatment can induce activation of PI3K/Akt in mouse colonic epithelium (containing extensive adenocarcinoma, foci of adenomatous and dysplastic changes, and normal adjacent epithelium) and the effect of dietary strawberries on the activation, we detected phosphorylation of PI3K, Akt, ERK and NFκB. As shown in Figure 3A,B, phosphorylation of PI3K was significantly reduced from 7.3 fold in mice fed control diet to 4.3-fold, 3.9-fold and 0.6-fold in mice fed 2.5%, 5% or 10% strawberries, respectively. Strawberries also significantly inhibited phosphorylation of Akt, ERK1/2 and p65 NFκB.

### 3.6. Strawberries Down-Regulate Expression of Proinflammatory Mediators

We measured mRNA expression of representative proinflammatory mediators by real-time PCR in the colonic epithelium. As shown in Figure 3C, dietary strawberries down-regulated the mRNA expression of TNF-α, IL-1β, IL-6, COX-2 and iNOS in mouse colon.

### 3.7. Strawberries Reduce PGE_2_ and Total Nitrite Productions

We evaluated the effect of dietary strawberries on COX-2 and iNOS activities by measuring prostaglandin E_2_ (PGE_2_; Figure 3D, upper panel) and total nitrate and nitrite levels (Figure 3D, lower panel). PGE_2_ production was reduced from 247 ± 13 pg mg^−1^ protein in mice treated with AOM/DSS only to 172 ± 15 (30% reduction; *p* < 0.05), 110 ± 10 (56% reduction; *p* < 0.01) and 75 ± 7 pg mg^−1^ (70% reduction; *p* < 0.01) protein in mice treated with AOM/DSS plus 2.5%, 5% or 10% strawberries, respectively. We evaluated the effects of strawberries on iNOS activity by measuring total nitrate and nitrite levels. The productions of total nitrate and nitrite were reduced by strawberries relative to animals fed control diet (*p* < 0.05).

## 4. Discussion

Despite advances in screening and early detection, colonoscopy, surgery and systemic chemotherapy, colon cancer remains a major cause of cancer death and a significant health care burden worldwide. In addition to environmental and food-borne mutagens, heritable genetic changes, chronic intestinal inflammation and lack of physical activity are risk factors for CRC. Research implicates an affluent dietary pattern characterized by excess caloric intake, rich in red and processed-meat, high saturated fat and low in fruits, vegetables and fiber [2]. This will allow researchers and clinicians to engage in targeted dietary interventions to inhibit colon carcinogenesis. A combination of pharmacologic and dietary interventions may have advantages of targeting diverse components of the carcinogenic process with less-overlapping toxicity and greater impact. The specific dietary and food components with anticancer activities remain to be clearly defined. In this study, we document the reduction of murine colon carcinogenesis in the AOM/DSS model by lyophilized strawberries. We further show that in this model of inflammation-driven cancer progression, dietary strawberries exhibit a down-regulation of several proinflammatory cytokines and suppression of oncogenic signaling.

The anticancer activity of strawberries is likely due to its pattern of nutrients and bioactives including vitamins (A, C, E and folate), minerals (selenium) and phytochemicals such as β-sitosterol, tannins (ellagitannins), flavonols (kaempferol, quercetin), stilbenes (resveratrol) and various anthocyanins [30]. These bioactives have been shown to have anti-inflammatory, antioxidant effects and involve in cellular metabolism, survival and other signaling pathways [31,32]. In this study, we identified that anthocyanins account for 58.4% by dry weight of the phenolics in the strawberry powder. The anthocyanin-rich extracts derived from other berry types, such as black raspberries, *Rubus croceacanthus* and bilberries, have been assessed for their anti-cancer potentials *in vitro* [33,34,35]. Pelargonidin-3-glucoside is the main anthocyanin in strawberries and reported to inhibit proliferation and invasion in prostate and colon cancer cells [36,37]. We note that pelargonidin malonyl glucoside is the secondary main anthocyanin in strawberries and counts for 9.38% of phenolics by dry weight. To our knowledge, its bioactive effect has not been assessed as a pure chemical substance, thus, it warrants further investigation of its effect *in vivo* and *in vitro*. We also identified agrimoniin, the main ellagitannin, as the second most abundant phenolic by dry weight in strawberries (16.16%). Agrimoniin and kaempferol are known to exhibit anti-inflammatory actions *in vitro* [38,39].

Bioavailability and pharmacokinetic studies of strawberry anthocyanins show that only modest amounts of ingested anthocyanins are absorbed from the upper small intestine. The bulk of phytochemicals enter the colon, where the substantial microbial metabolism and interaction with the colonic epithelium exists [40]. It is clear that the colonic microbiome impacts colon cancer risk. The pathogenesis, however, remain to be clearly elucidated [41]. It is possible that dietary anthocyanins my impact the colon microbiome. An alternative hypothesis is that the microbiome modulates anthocyanin metabolism and those metabolites are the most critical bioactives suppressing inflammation and colon carcinogenesis. It is also suggested that other dietary components may modulate the colonic microflora composition and metabolism, such as fermentable fiber, which suggests that a dietary pattern may be more strongly implicated in CRC prevention than single dietary variables.

Immune and inflammatory processes appear to be a common feature contributing to the genesis of human colon cancer. The murine model induced by the combination of a single injection of AOM followed by DSS, a potent proinflammatory stimulus, appears to be a consistent and reproducible model with histopathologic relevance to human disease, particularly mimicking features of IBD [42,43]. We observed that in parallel with suppression of carcinogenesis, a number of genes involved in the mucosal inflammatory response are down-modulated by dietary strawberries. NFκB plays an important role in connecting inflammation and cancer [44]. The activation of NFκB is regulated by several upstream kinases including PI3K/Akt and ERK [45]. Once activated, NFκB up-regulates the transcription of various proinflammatory mediators including TNF-α, IL-1β, IL-6, COX-2 and iNOS, which typically are overexpressed in colon cancer [46,47,48].

TNF-α, an important proinflammatory cytokine, can regulate other cytokines during inflammation responses [49]. Overexpression of TNF-α has been detected in human IBD. Infliximab, an antibody against TNF-α, has been shown to improve the symptoms of IBD in clinical trials [50]. IL-1β and IL-6 are proinflammatory cytokines and regulated by NFκB [51]. TNF-α, IL-1β and IL-6 play important roles in the tumor microenvironment and contribute to the promotion of inflammation associated carcinogenesis [52]. NO is endogenously generated from L-arginine by NOS. The association between chronic inflammation and cancer appears to be due, in part, to the production of high levels of NO and peroxynitrite. Nitrotyrosine is formed when peroxynitrite interacts with protein tyrosine and therefore, serves as a useful biomarker of nitrosative stress. Our study shows that the activation of nitortyrosine immunoreactivity following AOM/DSS treatment is inhibited by dietary strawberries.

A wealth of evidence derived from cell culture, rodent models and human investigations suggests that bioactive lipids participate in the colonic inflammatory cascade and carcinogenesis [53]. PGE_2_ is mainly generated from arachidonic acid by increased level of COX-2 in cancers. We measured PGE_2_ as a biomarker for a proinflammatory state in the colonic epithelium and found a dose-dependent inhibition of PGE_2_ production by dietary strawberry powder. The prostaglandin pathway has been targeted for colon cancer chemoprevention by steroids that inhibit arachidonic acid release, and by nonsteroidal anti-inflammatory agents (NSAIDs) that block COX-1 and COX-2 [54]. However, their use as a cancer preventive agent is restricted due to the potential toxicity and the incomplete understanding of the complex role in immune regulation and epithelial biology.

**Figure 4:**
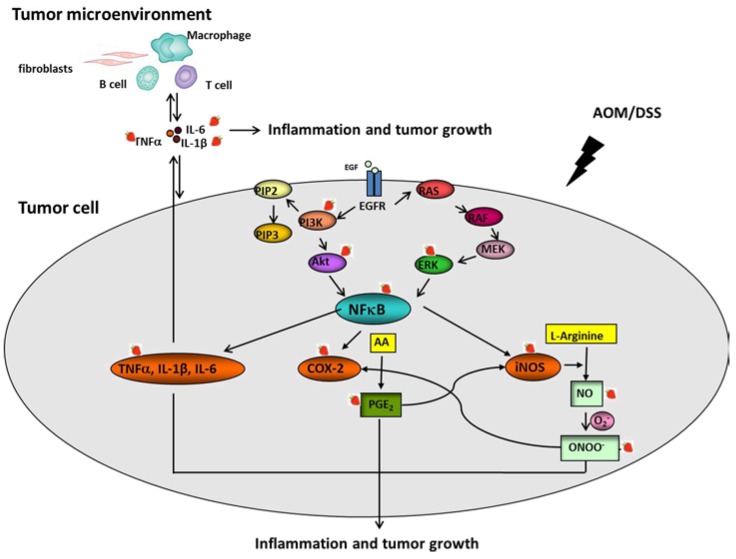
Possible interactive mechanisms of inhibition of AOM/DSS-induced colon cancer in mice by strawberries.

In the present study, we observed inhibitory effects of strawberries on proinflammatory mediators and related oncogenic signaling pathways. Our findings suggest that strawberries have a broad impact on multiple colonic inflammatory responses that are associated with colon carcinogenesis. In Figure 4, we depict interactive mechanisms of strawberries against AOM/DSS-induced tumor development in mouse colon.

## 5. Conclusions

To our knowledge, this is the first study to evaluate the efficacy of strawberries in inflammation-associated colon carcinogenesis *in vivo*. Whole foods, with a diverse array of bioactive phytochemicals, are predicted to suppress carcinogenesis through multiple and potentially interactive mechanisms of action. This study lays the foundation for testing of strawberries or strawberry food product(s) in future colon cancer prevention trials, and simultaneously provides leads for the development of specific phytochemicals or metabolites as chemopreventive agents using the principles of pharmacognosy.

## Supplementary Files

Supplementary File 1
