# Supplementary material for: Strawberry Phytochemicals Inhibit Azoxymethane/Dextran Sodium Sulfate-Induced Colorectal Carcinogenesis in Crj: CD-1 Mice
Source: Nutrients. 2015 Mar 10;7(3):1696–715. doi: 10.3390/nu7031696 (PMC4377876; doi:10.3390/nu7031696)
Supplement: Supplementary File 1 [file nutrients-07-01696-s001.docx]

**Supplementary Information**

**Table S1.** Primer Sequence for Real-time PCR.

| **Gene** | **Sense primer** | **Antisense primer** |
| --- | --- | --- |
| COX-2 | 5’-TGTATGCTACCATCTGGCTTCGG-3’ | 5’-GTTTGGAACAGTCGCTCGTCATC-3’ |
| iNOS | 5’-TTGGGTCTTGTTAGCCTAGTC-3’ | 5’-TGTGCAGTCCCAGTGAGGAAC-3’ |
| TNF-α | 5’-TACCTTGTCTACTCCCAGGTTCTC-3’ | 5’-AGAGCAATGACTCCAAAGTAGACC-3’ |
| IL-1β | 5’-CTCTTACTGACTGGCATGAGG-3’ | 5’-CCTTGTAGACACCTTGGTCTTGGAG-3’ |
| IL-6 | 5’-TAGCCGCCCCACACAGACAG-3’ | 5’-GGCTGGCATTTGTGGTTGGG-3’ |
| GAPDH | 5’-TATTGGGCGCCTGGTCACCA-3’ | 5’-CCACCTTCTTGATGTCATCA-3’ |

© 2015 by the authors; licensee MDPI, Basel, Switzerland. This article is an open access article distributed under the terms and conditions of the Creative Commons Attribution license (http://creativecommons.org/licenses/by/4.0/).
